# Depression in medical students: insights from a longitudinal study

**DOI:** 10.1186/s12909-017-1006-0

**Published:** 2017-10-10

**Authors:** Vanessa Silva, Patrício Costa, Inês Pereira, Ricardo Faria, Ana P. Salgueira, Manuel J. Costa, Nuno Sousa, João J. Cerqueira, Pedro Morgado

**Affiliations:** 1 Life and Health Sciences Research Institute (ICVS), School of Medicine, University of Minho, Braga, Portugal; 2 ICVS/3B’s, PT Government Associate Laboratory, Braga/Guimarães, Portugal

**Keywords:** Medical student, Distress, Depression, Anxiety, Burnout

## Abstract

**Background:**

Factors associated with depression of medical students are poorly understood. The purpose of this study is to determine the prevalence of depression in medical students, its change during the course, if depression persists for affected students, what are the factors associated with depression and how these factors change over time.

**Methods:**

A prospective, longitudinal observational study was conducted at the Medical School of the University of Minho, Portugal, between academic years 2009–2010 to 2012–2013. We included students who maintained their participation by annually completing a questionnaire including Beck Depression Inventory (BDI). Anxiety and burnout were assessed using the State Trait Anxiety Inventory and Maslach Burnout Inventory. Surveys on socio-demographic variables were applied to evaluate potential predictors, personal and academic characteristics and perceived difficulties. ANOVA with multiple comparisons were used to compare means of BDI score. The medical students were organized into subgroups by K-means cluster analyses. ANOVA mixed-design repeated measurement was performed to assess a possible interaction between variables associated with depression.

**Results:**

The response rate was 84, 92, 88 and 81% for academic years 2009–2010, 2010–2011,2011-2012 and 2012/2013, respectively. Two hundred thirty-eight medical students were evaluated longitudinally. For depression the prevalence ranged from 21.5 to 12.7% (academic years 2009/2010 and 2012/2013). BDI scores decreased during medical school. 19.7% of students recorded sustained high BDI over time. These students had high levels of trait-anxiety and choose medicine for anticipated income and prestige, reported more relationship issues, cynicism, and decreased satisfaction with social activities. Students with high BDI scores at initial evaluation with low levels of trait-anxiety and a primary interest in medicine as a career tended to improve their mood and reported reduced burnout, low perceived learning problems and increased satisfaction with social activities at last evaluation. No difference was detected between men and women in the median BDI score over time.

**Conclusions:**

Our findings suggest that personal factors (anxiety traits, medicine choice factors, relationship patterns and academic burnout) are relevant for persistence of high levels of BDI during medical training. Medical schools need to identity students who experience depression and support then, as early as possible, particularly when depression has been present over time.

## Background

Despite an increasing interest in distress during medical training, factors associated with medical student distress, particularly depression, are poorly understood. Mental health care of medical students is a complex process influenced by innate characteristics of the student, training-related stressors [1] and many other factors. In medical students, the prevalence of depression ranged between 2.9 and 38.2% [2, 3] and this prevalence is similar or higher compared to that of the general population. [4–6] In Portugal the overall rate of depression ranges from 6.1 to 21.5% [7, 8]. Most authors suggest that depression increases during medical training [4, 9, 10] and this increase is more pronounced in women [4, 11]. However, the rise of depression during medical training is not universally reported: studies show that depression decreases from the first to the second year of medical training, and between preclinical and clinical years [12, 13]. Indeed, *Dyrbye* et al. [9] suggest that this prevalence varies depending on the age of medical students, the stage of medical training, the methodology for evaluating depression and location. The use of study instruments that limit the extent of direct comparison of medical students to similarly aged members of the population lies at the heart of this.

Medical student depression was linked to substance abuse [14], suicide and impaired professional function [15, 16], interpersonal skills [17–19], professionalism [20–22], physical and mental health [16, 23].

The relationship between the development of depression and the greater future risk of recurrence of depressive episodes and long-term morbidity is consensual [24]. This finding can have impact not only during the doctor’s pre-graduate training but also after this, thus affecting patients, given the link between physician depression and a decrease in the quality of health care provided by increasing cynicism, poor communication, and rising of medical errors [25]. Medical well-being should be the precursor to physician well-being and a way to enhancement of professionalism and patient care, and these findings bring this critical problem in graduate medical education to light.

Longitudinal studies have suggested that some students experience recurrent episodes of depression during medical education [26, 27], but not many studies have investigated whether depression is persistent, for a particular student, and what factors are associated with persistence, recovery, and resilience to depression during medical school. Previous studies have identified personality traits as components of the medical student’s coping reservoir and as predictors for outcomes of mental health [28, 29]. Indeed, Bunevicius et al. [30] suggest that anxiety is inversely proportional to emotional stability and straight proportional to the vulnerability to stress, for medical students.

The stress of medical school drains the coping reservoir but social and health beneficial activities may replenish it [28]. Medical students with small coping reservoir or few positive inputs are at greater risk of distress, including burnout [28]. An examination of burnout literature reveals that it is prevalent in medical students (28–45%) [31], and depression and burnout seem to be closely linked [26]. This might reflect the same vulnerability and a close relationship between these entities.

Given the importance of depression and its consequences, it is important to assess its temporal unfolding along the student’s path in medical school to identify its critical period and disclose whether other conditions could also be linked to this phenomenon. Prospective longitudinal data also allows an analysis of long-term predictors and identification and characterization of vulnerable and resilient students. This finding will help to design a basis for adequate preventive measures and strategies to deal with depression and its consequences, and for establishing healthy studying conditions. The promotion of mental health by medical schools, can be achieved through the recognition and appreciation of the individual characteristics and psychological function of the medical student. [28]. This will bring benefits to the medical education sector, the medical industry, increase medical professionals’ resilience and satisfaction, and improve the quality of care for patients.

The avoidance of professional help and treatment by medical students [26, 32] increases the responsibility of medical schools in identifying and understanding the prevalence of depression and its underlying factors [28]. This study aims to contribute to the comprehension of depression in medical students, with the certainty that this knowledge will be useful to aid in the design of preventive strategies and more effective interventions to improve the quality of medical education. For this, we answer some questions that concerns medical schools and students.

1. What is the prevalence of depression?
2. How does the prevalence of depression change during the degree?
3. Is depression persistent for those affected?
4. What personal and academic factors are associated with persistence of depression? And with the recovery?
5. Do these factors change over time?

## Methods

### Study design and setting

An observational, longitudinal and prospective study was designed at Medical School - University of Minho.

### Participants

All, including foreign, students in the 2009–2010, 2010–2011, 2011–2012 and 2012–2013 cohorts of medical students at Medical School - University of Minho were invited to participate. Medical students participated in the study without incentives and there were no exclusion criteria.

### Procedures

Printed copies of the questionnaires and a confidentiality disclaimer were administered by the senior year student at the end of regular classes during the October month for all students in the 2009–2010, 2010–2011, 2011–2012 and 2012–2013 academic year, in a selected time without stressful study periods or examinations. Each student completed the questionnaire including Beck Depression Inventory (BDI), State Trait Anxiety Inventory (STAI), Maslach Burnout Inventory - student version (MBI-SS), a socio-demographic and perceived difficulties questionnaire. The questionnaire was completed anonymously and returned in ballots. Only an individual identification code was provided to allow longitudinal data analysis. Ethics approval was obtained for this research from the Ethics Subcommittee for the Life Sciences and Health of the Medical School – University of Minho, Portugal.

### Outcomes

We measured depression using the BDI created by Beck [33]. It is a 21-question multiple-choice self-application instrument, one of the most used psychometric scales for assessing the severity of depression. It was adapted to the Portuguese population by Vaz Serra [34, 35]. The cutoff points were proposed by Ribeiro et al. [36], identifying individuals as not depressed (0–12 points) and depressed (≥13 points).

### Potential predictors

Age, gender, sexual orientation, motivation to enroll in the medical degree and residence were collected as sociodemographic characteristics. Academic variables such as the year of study, academic performance, and curricular year were also surveyed. Items covering recent (previous 6 months) perceived difficulties or problems with substance abuse, skills/organization, learning, relationship, health, psychological help, social support, and adverse life events were included. These factors were found to potentially predict the mental health outcomes and may thus influence the coping reservoir [28].

Anxious trait was reported using the subscale Trait Anxiety Scale (T-Anxiety) of the STAI. This self-application instrument, with 20 items, developed by Spielberger [37] was adapted to the Portuguese population by Danilo Silva [38]. T-Anxiety is based on individual differences in propensity for anxiety and assess the individual “usually feels”, varying the response from “almost never” to “almost always”. The score varies between 20 and 80 and higher scores correspond to a more pronounced anxious trait. To identify individuals with high trait-anxiety, we used the percentile 75 as the cutoff point. Burnout in its three dimensions: emotional exhaustion, cynicism and academic inefficiency in the student population were assessed through MBI-SS. The MBI-SS is the standard self-application tool with 15 items developed by Shaufeli [39] and the version used for this study was translated into Portuguese by Faria et al. with high reliability [8]. MBI-SS was validated to the Portuguese population by João Maroco [40]. Individuals with high burnout symptomatology were identified using the 75 percentile according to the recommendations of MBI-SS guidelines [39]. In this study, we chose to use the term academic inefficiency, measured by the inversion of items related to academic effectiveness.

### Analysis

#### Overall approach

To assess the prevalence of depression, we included in this study all students completing questionnaires between 2009–2010 to 2012–2013 academic year.

To answer the remaining questions, we included only students who maintained their participation in all moments of the study, tracking by their individual identification code.

To assess if the prevalence of depression increases or decreases during the course we grouped all students in classes, named them by the year of beginning of medical school (Class 07, Class 08, and Class 09), using the academic track – study year. It should be noted that in this group of students there were no students who have not carried over from academic year during the study.

### Statistical analysis

Statistical analysis was performed using the Statistical Package for the Social - Sciences (SPSS) version 20.0 for Windows. Univariate analysis was used for descriptive statistics (absolute and relative frequency, mean (M), standard deviation (SD), maximum and minimum). A bivariate analysis was used to test differences in BDI scale between groups (t-test, one-way ANOVA with multiple comparisons by post-hoc Tukey-HSD for independent samples, ANOVA for repeated measures using the correlation of Boneferroni for paired samples and ANOVA for repeated measures mixed-design). Multivariate analysis with K-means method was used to cluster the sample according to BDI scores. We set the level of statistical significance at 5% (*p* < 0.05) for all analyses.

## Results

### Participants

The average response rate was 86% (*n* = 2234). In the longitudinal analysis we included students who participated each year and provided depression data (238 medical students – 74%).

### Prevalence of depression

Table 1 demonstrates that the prevalence of depression ranged between 12.7 and 21.5%. The prevalence of suicidal thoughts ranged from 2.6 to 6.2% (2009–2010 and 2012–2013 academic year, respectively). Fatigue and sleep problems were the most reported symptoms (55.9% - 74% and 49.4% - 65.4%, respectively) in the same academic years.

**Table 1:** Descriptive analysis of BDI scores - absolute frequencies (*n*) and relative frequencies (%)

		*n* (%)			
		2009-2010	2010-2011	2011-2012	2012-2013
BDI Score	0-12 points	365 (78.5)	461 (82.2)	504 (86.9)	548 (87.3)
	≥13 points	100 (21.5)	100 (17.8)	76 (13.1)	80 (12.7)

### Prevalence of depression over time

There was a statistically significant downward trend in the average scores of the depression inventory in the first 3 years of the study (2009–2010: M = 8.57, SD = 7.13; 2010–2011: M = 7.13, SD = 6.47; 2011–2012: M = 6.08, SD = 6.35).

**Fig. 1:**
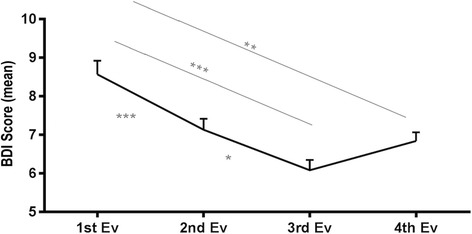
BDI score for school year and representation of significant differences obtained by means of multiple comparisons using the Bonferroni correlation (*p < 0.05, **p < 0.01, ***p < 0.001)

In the 4th year this trend was reversed by a non-significant increase in their average values (2012–2013: M = 6.84, SD = 6.65). This is graphically demonstrated in Fig. 1.

**Fig. 2:**
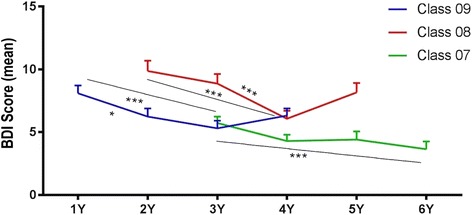
BDI score for class and school year with representation of significant differences obtained by means of multiple comparisons using the Bonferroni correction (*p < 0.05, **p < 0.01, ***p < 0.001)

As can be gleaned from Fig. 2, there was a statistically significant downward trend in the mean BDI scores of each cohort across the years, as the cohorts’ progress through the medical degree and median BDI score is statistically different for each academic year between the different classes.

### Persistence of depression

**Fig. 3:**
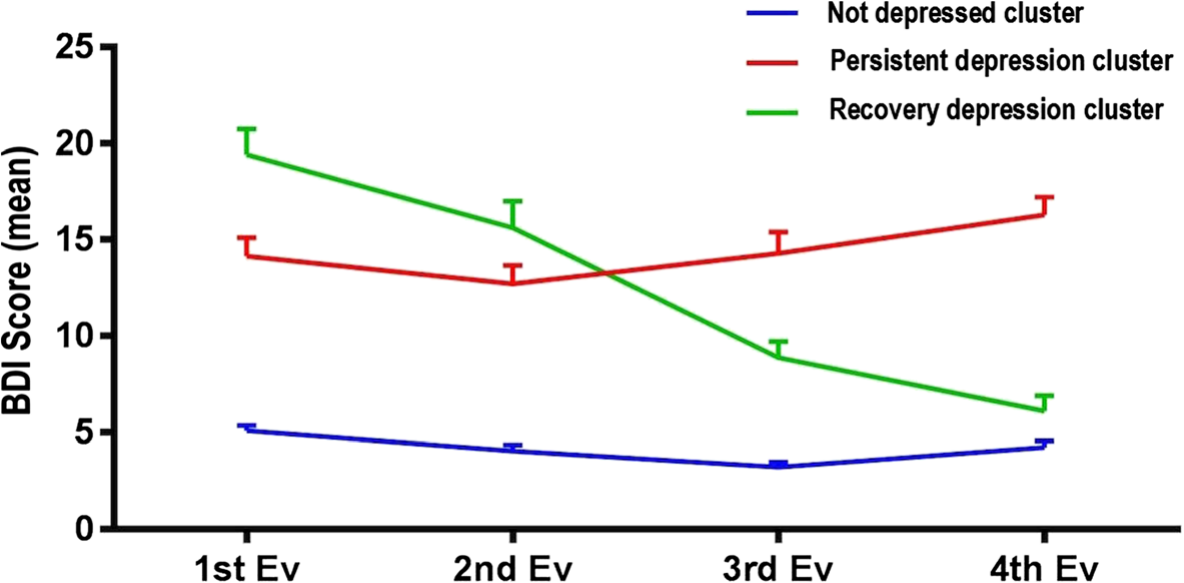
Analysis cluster. BDI mean depending of the BDI result in the 4 evaluations, by “K-means” method

The K-means method was used to cluster the sample according to the BDI scores. The analysis showed three clusters, as seen in Fig. 3. Indeed, cluster 1 (not depressed cluster - 68.5%) presents sustained low mean BDI scores over the years of study. Cluster 2 (persistent depression cluster - 19.7%) presents a sustained mean of BDI scores representing moderate depression that persists over time. On the other hand, cluster 3 (recovery depression cluster - 11.8%) begin with mean of BDI scores representing moderate depression at the start of their undergraduate career and this mean BDI scores decrease during the program, up to values ​​that indicate absence of depression.

These three clusters are statistically different from each other.

### Personal and academic factors associated with persistent depression

The intersection with dimensions associated with depression allowed quantitatively describes the clusters, as we can see on Table 2 and below.

- **Not depressed cluster** - presents low levels of trait-anxiety associated with low burnout scores. In this cluster students are satisfied with the network and social activities, report low perceived learning problems, low perceived relationship problems, low perceived health problems, and a primary interest in medicine.
- **Persistent depression cluster** - presents high levels of trait-anxiety, dissatisfaction with academic ratings. Many had perceived learning problems. Anticipated income and prestige were crucial to choosing medicine for the majority of the members of this cluster.
- **Recovery depression cluster** - presents low levels of trait-anxiety and most students report a primary interest in medicine. In this cluster students are satisfied with the network and with academic achievement.

**Table 2:** Characterization of clusters (absolute and relative frequencies) and analysis of depression clusters per dimensions associated with depression (one-way ANOVA)

			Not depressed cluster		Persistent depression cluster		Recovery depression cluster		
			n	%	n	%	n	%	F(2,237)
Sex	Female		110	67.5	34	72.3	25	89.3	2.81
Trait-anxiety	Yes		16	9.8	28	59.6	6	21.4	66.48***
*Burnout*	Emotional exhaustion	Yes	16	9.8	22	46.8	16	57.1	31.19***
	Cynicism	Yes	14	8.6	15	31.9	15	53.6	23.09***
	Academic ineffectiveness	Yes	33	20.2	18	38.3	14	50.0	7.47**
Motivation to enroll in the medical degree	Vocational interest	Yes	131	80.4	11	31,9	23	82.1	1.76**
	Professional security	Yes	32	19.6	36	68.1	5	17.9	2.58*
Place of residence	Household	Yes	93	57.1	16	34.0	11	39.3	4.78**
Problems / difficulties	Substance abuse	Yes	7	4.3	5	10.6	2	7.1	1.37
	Skills / organization	Yes	131	80.4	42	89.4	27	96.4	2.08
	Learning	Yes	61	37.4	36	76.6	23	82.1	20.37***
	Relationship	Yes	32	19.6	16	34.0	14	50.0	7.01**
	Health	Yes	39	23.9	20	42.6	22	78.6	19.37***
Social support - Satisfaction	Social network	Yes	154	94.5	21	78.7	23	82.1	13.66***
	Social activities	Yes	149	91.4	31	66.0	12	42.9	26.84***
Academic achievement	Academic ratings	Yes	100	61.3	12	25.5	8	71.4	6.30**

(*p < 0.05, **p < 0.01, ***p < 0.001)

### Interaction between variables associated with depression over time

ANOVA mixed-design repeated measurement was performed to assess a possible interaction between variables associated with depression, in the 1st and 4th assessment. Clusters and the t-test were used in paired samples to decompose interactions. Below we present only the variables that show an interaction. There is interaction between clusters and the following variables in the 1st and 4th data collection point:

- **MBI - emotional exhaustion**: F (2,238) = 7.85, *p* < 0.01, ηp^2^ = 0.06. Decomposing interactions: there are significant differences between the not depressed cluster and the recovery depression cluster (M_1_ = 11.57, SD = 4.03; M_4_ = 10.60, SD = 4.30, *p* < 0.05, and M_1_ = 17.43, SD = 5.34, M_4_ = 12.64, SD = 3.37, *p* < 0.001, respectively). Thus for the not depressed cluster and the recovery depression cluster there is a significant reduction in emotional exhaustion over time.
- **MBI – cynicism**: F (2,238) = 11.37, *p* < 0.001, ηp^2^ = 0.09. Decomposing interactions: there are significant differences between the persistent depression cluster and the recovery depression cluster (M_1_ = 6.30, SD = 4.01; M_4_ = 8.04, SD = 3.93, *p* < 0.05, and M_1_ = 8.36, SD = 4.82; M_4_ = 5.79, SD = 2.85, *p* < 0.01, respectively). Thus for the persistent depression cluster there is a significant increase, and for the recovery depression cluster a significant decrease in cynicism over time.
- **MBI - academical inefficacy**: F (2,238) = 12.26, *p* < 0.05, ηp^2^ = 0.03. Decomposing interactions: there are significant differences in the recovery depression cluster (M_1_ = 17.54, SD = 3.73; M_4_ = 15.50, SD = 4.22; *p* < 0.05). Thus for the recovery depression cluster there is a significant decrease in academic ineffectiveness over time.
- **Learning problems**: F (2,238) = 4.02, *p* < 0.05, ηp^2^ = 0.03. Decomposing interactions: there are significant differences in the not depressed cluster and recovery depression cluster (M_1_ = 0.37, SD = 0.48, M_4_ = 0.19, SD = 0.34, *p* < 0.001 and M_1_ = 0.82, SD = 0.39, M_4_ = 0.43, SD = 0.50, *p* < 0.01, respectively). Thus for the not depressed cluster and the recovery depression cluster there is a significant decrease in learning problems over time.
- **Relationship problems**: F (2,238) = 4.48, *p* < 0.05, ηp^2^ = 0.03. Decomposing interactions: there are significant differences in the persistent depression cluster (M_1_ = 0.34, SD = 0.48, M_4_ = 0.55, SD = 0.50; *p* < 0.05). Thus for the persistent depression cluster there is a significant increase in relationship problems over time.
- **Satisfaction with social activities**: F (2,235) = 4.87, *p* < 0.001, ηp^2^ = 0.09. Decomposing interactions: there are significant differences between the persistent depression cluster and the recovery depression cluster (M_1_ = 0.66, SD = 0.48, M_4_ = 0.43, SD = 0.50, p < 0.05, M_1_ = 0.43, SD = 0.50, M_4_ = 0.75, SD = 0.44, p < 0.01, respectively). Thus for the persistent depression cluster there is a significant decrease, and for the recovery depression cluster a significant increase, in satisfaction with social activities over time.

## Discussion

The results indicated that 12.7 to 21.5% of the responding medical students had clinical depression. These results are consistent with the literature [2–6, 9] and higher than those of the general population and their peers [9]. Studies that used BDI for measuring depression in medical students in other countries produced similar results [6, 41–43].

Recently, Coentre et al. [7] report lower prevalence (6.8%) of depression among medical students in Portugal than ours. The cut-off of BDI scale for depression (13) used in this study may have contributed to a higher prevalence of depression in our participants.

Although the prevailing literature suggests that depression worsens with academic training [1, 4, 10], a statistically significant decrease in the mean BDI score was found in this study. These findings reinforce other studies [12, 13] and open up new perspectives on this theme, showing that for the studied population, depression improves with academic training. The causal factors are not clear, and this difference may be explained in part by the adaptation to the demands of the course, maturing of participants, institutional changes or external factors.

Looking at the analysis per entrance cohort, we inferred that depression levels vary with the academic year and with the group of students that attend each year. The prevalence of depression is not maintained throughout the school years, so the demand of an academic year of medical school cannot seem to explain entirely the prevalence of depression in the study group. On the other hand, Class 07 keeps in four evaluations with lower BDI mean scores, whereas Class 08 features high scores for depression in 2 of 4 evaluated years. Interactions obtained between class and BDI scores suggest that differences in class environment (cooperative learning and peer support) have more impact on BDI scores than curriculum. We supposed that “class effect” has more relevance than the “effect of the academic year”, but more studies are needed. Our study is one of few that examined persisting depression in students over time. We designed this study to recognize medical students resilient to depression and uncover how these students differed from their peers. We found a group of students who exhibited stably low levels of depression. Compared to the group with persistent mild levels of depression over time (the persistent depression cluster), the stable students (the not depressed cluster) present low levels of trait-anxiety, primary interest in medicine, low burnout scores, low perceived learning problems and increased satisfaction with social activities. All these factors have already been mentioned in several studies as protective factors for depression in medical students [19, 44, 45]. Literature shows that students pressed to select the medical profession are more prone to depression [45] and medical students with higher perceived levels of life satisfaction consider that medical training have less negative impact on their personal and social lives and were less likely to use maladaptive strategies such as emotion focused copping, compared to their peers [44].

On the other hand, high levels of trait-anxiety, anticipated income and prestige to choose medicine, higher perceived learning problems, and decreased satisfaction with social activities were important factors to maintaining depression (the persistent depression cluster). The fact that that women experienced persistent depression more likely than men was not clear from our results, when compared to other clusters in this study.

Many students experienced depression as a transient state. Despite the small size of the cluster sample which may condition the extrapolation of results, the recovery depression cluster begins with mean of BDI scores representing moderate depression at the start of their undergraduate career and this mean BDI scores decrease during the program, up to values ​​that indicate absence of depression. This cluster present low levels of trait-anxiety and primary interest in medicine seem to favor depression remission. These results show the importance of personality, namely anxiety trait, in vulnerability and evolution of depression [46–48] and the influence of vocational factors in psychological stability and adaptive capacity [45].

Medical students able to overcome depression show decrease in burnout levels and the perception of learning problems and increased satisfaction with social activities over time. Despite being depressed at earlier stages of the course these students seem to have better adapted to the demands of the course with decreased learning problems and academic inefficiency levels. They seem to be able to establish a healthier balance between academic activities and the remaining areas of their life, with increased satisfaction with social activities, decreased emotional exhaustion and unconscious distancing from the studies. The interaction between these factors influences the evolution of depression, as reported in several other studies [9, 19, 26, 48] and should be explored by medical schools.

Students depressed throughout the course show increased perception of relationship problems, cynicism, and decreased satisfaction with academic activities. This sacrificial behavior has been described to potentiate a vicious cycle that impairs professional function [15], interpersonal skills [17–19], professionalism [20–22], and physical and mental health [16, 23]. Despite the persistence of depression being low these is a crucial group of students who continuously experience depression.

Students of medicine are required to learn a vast amount of material in a small frame of time, which leads to a lot of stress. Their “coping reserve” is constantly at risk of significantly dwindling because they have less time and energy to form and sustain relationships and for self-care [7]. This can partially explain medical students’ vulnerability to depression. Medical schools would help depressed students, particularly those whose depression is persistent, by forming a system that identifies and supports as early as possible, because evidence suggests that these students were reluctant to seek help [41, 49, 50]. All intervention measures must be designed according to the needs of this important group of students requiring a removal of barriers by clearing the stigma linked to searching mental health treatment. Medical schools should also promote students to anonymously access external mental health care. Medical schools need to implement student wellness programs providing opportunities and resources for healthy life. These programs could encouraged students to cultivate empathy, stress management, professionalism, frustration tolerance, and active strategies of coping, self-care based on self-diagnosis of depression, knowledge of life contexts, academic and personal practices, maintaining and creating interests that go beyond medicine, for example regular physical activity. The positive effects of exercise on well-being and mental health are extensively documented [51]. A good start for medical school promoting mental health can be leave space to regular physical activity in the curriculum [13, 52]. Mentorship is another crucial pillar helping students’ professional and personal development [28]. A mentorship system involving faculty and senior students, seems to be beneficial by helping medical students to manage academic training and life stressors [53–55]. Oher interventions, including strategies to develop cognitive restructuring and problem solving [25, 46], life skills training and mindfulness therapy [56, 57], individual counselling [28], adaptive and communication skills training [25] and social support [3] can also be offered as part of the curriculum.

This study strengthens the need that institutions recognize that the population of medical students is vulnerable to depression and other mental health illnesses. We must reflect and act, because depression in medical students has personal cost to them, future costs to organizations, and health care costs to patients.

### Strengths and limitations

The prospective and longitudinal design of this study is its greatest strength. However, the single-center nature of the design can limited the generalizability of our results. In addition, the absence of a control group of students who do not attend medical school is also a limitation of this study. Even though the number of responses over the 4 years of the study was high, some datasets could not be matched as they were identified by incorrect individual identification codes. These participation rate, associate to the fact later non-responder cannot be predicted by initial scores (by missing data analyses) support the generalizability of our results to the entire body of medical students at the Medical School of the University of Minho. We must take into account the possibility of a link between the missing data and an unobserved change in depression or students who did not participate in the study.

The use of self-application instruments instead of clinical structured interviews could be a limitation. Although used in several studies [41, 48, 53, 58], mean BDI scores may not reflect the severity of depression, which can be interpreted as a limitation of the present study.

The strong comorbidity of depression, anxiety, and burnout was not taken into consideration. This study did not explore how the presence of one dimension of distress determines the presence or absence of another.

Studies have shown that students with prior mental health problems [49] or personality aspects beyond trait anxiety, such as maladaptive perfectionism [53] can be linked to depression. More comprehensive study is needed about the predictors of depression in medical students, including individual factors, such as physical examination parameters, biological (like salivary cortisol levels), genetic and sociodemographic characteristics [25]. Personality factors, contextual factors like external stressors, learning conditions or life events should also be comprise [25].

## Conclusion

Despite decreasing during medical training, some students continuously experience depression. Our findings suggest that personal factors (anxiety traits, medicine choice factors, relationship patterns and academic burnout) are relevant for maintenance of high levels of depression during medical training. Installing procedures that identify and support depressed students, specially the few with persistently low mood is crucial.

It is vital to clean up the mental health-related stigma among health professionals to promote students having trouble seeking and receiving appropriate help.

This finding justifies the implementation of specific preventive programs to foment resilience and individual fulfillment and for improvement of patient care and mental resilience. This would suit the requirement of future physicians and the interest of patients and the community in the long term.

## Abbreviations

BDI: Beck depression inventory
MBI-SS: Maslach burnout inventory
STAI: State trait anxiety inventory
